# Potent α-amylase inhibitory activity of Indian Ayurvedic medicinal plants

**DOI:** 10.1186/1472-6882-11-5

**Published:** 2011-01-20

**Authors:** Sudha P, Smita S Zinjarde, Shobha Y Bhargava, Ameeta R Kumar

**Affiliations:** 1 Institute of Bioinformatics and Biotechnology, University of Pune, Pune 411 007, India; 2 Molecular Embryology Laboratory, Department of Zoology, University of Pune, Pune 411 007, India

## Abstract

**Background:**

Indian medicinal plants used in the Ayurvedic traditional system to treat diabetes are a valuable source of novel anti-diabetic agents. Pancreatic α-amylase inhibitors offer an effective strategy to lower the levels of post-prandial hyperglycemia via control of starch breakdown. In this study, seventeen Indian medicinal plants with known hypoglycemic properties were subjected to sequential solvent extraction and tested for α-amylase inhibition, in order to assess and evaluate their inhibitory potential on PPA (porcine pancreatic α-amylase). Preliminary phytochemical analysis of the lead extracts was performed in order to determine the probable constituents.

**Methods:**

Analysis of the 126 extracts, obtained from 17 plants (*Aloe vera (L.) Burm.f., Adansonia digitata L., Allium sativum L., Casia fistula L., Catharanthus roseus (L.) G. Don., Cinnamomum verum Persl., Coccinia grandis (L.) Voigt., Linum usitatisumum L., Mangifera indica L., Morus alba L., Nerium oleander L., Ocimum tenuiflorum L., Piper nigrum L., Terminalia chebula Retz., Tinospora cordifolia (Willd.) Miers., Trigonella foenum-graceum L., Zingiber officinale Rosc.*) for PPA inhibition was initially performed qualitatively by starch-iodine colour assay. The lead extracts were further quantified with respect to PPA inhibition using the chromogenic DNSA (3, 5-dinitrosalicylic acid) method. Phytochemical constituents of the extracts exhibiting≥ 50% inhibition were analysed qualitatively as well as by GC-MS (Gas chromatography-Mass spectrometry).

**Results:**

Of the 126 extracts obtained from 17 plants, 17 extracts exhibited PPA inhibitory potential to varying degrees (10%-60.5%) while 4 extracts showed low inhibition (< 10%). However, strong porcine pancreatic amylase inhibitory activity (> 50%) was obtained with 3 isopropanol extracts. All these 3 extracts exhibited concentration dependent inhibition with IC_50 _values, *viz*., seeds of *Linum usitatisumum *(540 μgml^-1^), leaves of *Morus alba *(1440 μgml^-1^) and *Ocimum tenuiflorum *(8.9 μgml^-1^). Acarbose as the standard inhibitor exhibited an IC_50 _(half maximal inhibitory concentration)value of 10.2 μgml^-1^. Phytochemical analysis revealed the presence of alkaloids, tannins, cardiac glycosides, flavonoids, saponins and steroids with the major phytoconstituents being identified by GC-MS.

**Conclusions:**

This study endorses the use of these plants for further studies to determine their potential for type 2 diabetes management. Results suggests that extracts of *Linum usitatisumum*, *Morus alba *and *Ocimum tenuiflorum *act effectively as PPA inhibitors leading to a reduction in starch hydrolysis and hence eventually to lowered glucose levels.

## Background

DM (Diabetes mellitus) is a metabolic disorder characterized by chronic hyperglycemia or increased blood glucose levels with disturbances in carbohydrate, fat and protein metabolism resulting from absolute or relative lack of insulin secretion [1]. The frequency of this disorder is on the rise globally, is likely to hit 300 million by 2025 with India projected to have the largest number of diabetic cases [2].

Type 2 diabetes is one of the primary threats to human health due to increasing prevalence, chronic course and disabling complications [3]. Many diverse therapeutic strategies for the treatment of Type 2 diabetes are in use. The conventional available therapies for diabetes include stimulation of endogenous insulin secretion, enhancement of the action of insulin at the target tissues, oral hypoglycemic agents, such as biguanids and sulfonylureas and the inhibition of degradation of dietary starch by glycosidases such as α-amylase and α-glucosidase [4].

Pancreatic α-amylase (E.C. 3.2.1.1) is a key enzyme in the digestive system and catalyses the initial step in hydrolysis of starch to a mixture of smaller oligosaccharides consisting of maltose, maltotriose, and a number of α-(l-6) and α-(1 - 4) oligoglucans. These are then acted on by α-glucosidases and further degraded to glucose which on absorbtion enters the blood-stream. Degradation of this dietary starch proceeds rapidly and leads to elevated PPHG (post-prandial hyperglycemia). It has been shown that activity of HPA (human pancreatic α-amylase) in the small intestine correlates to an increase in post-prandial glucose levels, the control of which is therefore an important aspect in treatment of type 2 diabetes[5]. Hence, retardation of starch digestion by inhibition of enzymes such as α-amylase plays a key role in the control of diabetes. Inhibitors of pancreatic α-amylase delay carbohydrate digestion causing a reduction in the rate of glucose absorption and lowering the post-prandial serum glucose levels [6]. Some inhibitors currently in clinical use are acarbose and miglitol which inhibit glycosidases such as α-glucosidase and α-amylase while others such as and voglibose inhibit α-glucosidase. However, many of these synthetic hypoglycemic agents have their limitations, are non-specific, produce serious side effects and fail to elevate diabetic complications. The main side effects of these inhibitors are gastrointestinal *viz*., bloating, abdominal discomfort, diarrhea and flatulence [7]. Herbal medicines are getting more importance in the treatment of diabetes as they are free from side effects and less expensive when compared to synthetic hypoglycemic agents [8,9].

In India, indigenous herbal remedies such as Ayurveda and other Indian traditional medicine have since ancient times used plants in treatment of diabetes[10]. Ethnobotanical studies of traditional herbal remedies used for diabetes have identified more than 1,200 species of plants with hypoglycemic activity [3,11]. A number of medicinal plants and their formulations are used for treating diabetes in the traditional Indian Ayurvedic system as well as in ethnomedicinal practices. Even though, these traditional practices are empirical in nature, over 200 million people in India with limited access to primary healthcare centres, depend on traditional system of medicine to cater to their healthcare needs [12]. However, this traditional knowledge, derived empirically, has to be supported by scientific testing. WHO (World Health Organization) (1980) has recommended the evaluation and mechanistic properties of the plants effective in such systems [13,14]. The search for new pharmacologically active agents obtained by screening natural sources such as medicinal plants or their extracts can lead to potent and specific inhibitors for α-amylase [6]. Pharmacological properties α-glucosidase inhibitors such as acarbose that can also inhibit pancreatic α-amylase revealed that the complications of DM such as onset of renal, retinal, lens and neurological changes and the development of ischaemic myocardial lesions are prevented or delayed [15]. Long-term day-to-day management of diabetes, with acarbose is well tolerated and can improve glycaemic control as monotherapy, as well as in combination therapy [16].

The Western Ghat area in the western region of India was declared an ecological hotspot in 1988 by the Government of India. Though this area covers barely five percent of India's land, 27% of all species of higher plants in India are found here. Some plants found here such as *Aloe vera (L.) Burm.f., Adansonia digitata L., Allium sativum L., Casia fistula L., Catharanthus roseus (L.) G. Don., Cinnamomum verum Persl., Coccinia grandis (L.) Voigt., Linum usitatisumum L., Mangifera indica L., Morus alba L., Nerium oleander L., Ocimum tenuiflorum L., Piper nigrum L., Terminalia chebula Retz., Tinospora cordifolia (Willd.) Miers., Trigonella foenum-graceum L., Zingiber officinale Rosc*. are well known in Ayurveda to possess anti-diabetic properties [8,9,17-21]. These plants are known to lower blood glucose levels and their traditional uses are listed in Table 1. So far, reports on the systematic evaluation and scientific investigation of these ant-diabetic plants or their phytoconstituents pancreatic α-amylase inhibitors are scarce. This study was carried out not only to validate the traditional uses of these plants in diabetes but also to initiate search for newer pharmacophores with specificity towards pancreatic α-amylase. Structurally as well as mechanistically, PPA (Porcine pancreatic α-amylase) is closely related to HPA (Human pancreatic α-amylase) [22]. Hence, sequential solvent extracts of the abovementioned plants were screened for the presence of PPA inhibitors, the lead extracts quantified for PPA inhibition under *in vitro *conditions and subjected to phytochemical analysis in order to identify the probable inhibitory phytoconstituents.

**Table 1:** Plant sources and their traditional uses

Plants Name	Family	Parts used	Hypoglycemic and medicinal properties	**Ref**.
*Adansonia digitata L*.	Bombacaceae	Leaves	Lowers blood glucose level due to insulin like effect on peripheral tissues; by promoting glucose uptake and metabolism or by inhibiting hepatic gluconeogenesis	[18]
*Allium sativum L*.	Alliaceae	Rhizomes	Lowers blood pressure and improves lipid profile, decreases serum glucose, triglycerides, cholesterol, urea, uric acid, increases serum insulin levels	[40]
*Aloe vera (L.) *Burm.f.	Liliace	Leaf Gel	Hypoglycemic activity, decreases fasting glucose levels, hepatic transaminases, plasma and liver cholesterol, triglycerides, free fatty acids and phospholipids. Improves plasma insulin level. Restores normal levels of LDL and HDL and cholesterol Reduces levels of hepatic phosphatidylcholine hydroperoxide and have hypocholesterimic efficacy, diminishes degenerative changes observed in kidney tissues	[41]
*Casia fistula L*.	Caesalpiniaceae	Leaves	Hypoglycemic activity decreases blood glucose level	[19]
*Catharanthus roseus (L.)*. G. Don	Apocynaceae	Leaves	Reduces blood glucose by enhancing secretion of insulin from β-cells of Langerhans or through extra pancreatic mechanism	[42]
*Cinnamomum verum *Persl.	Lauraceae	Bark	Reduces the blood glucose and elevates the plasma insulin level.	[43]
*Coccinia grandis (L.) *Voigt.	Cucurbitacea	Fruit	Reduces blood glucose and glycosylated hemoglobin content. *C. indica *extracts lowers blood glucose by depressing its synthesis, depression of glucose 6-phosphatase and fructose1,6, bisphosphatase and enhancing glucose oxidation by shunt pathway through activation of iglucose 6-phosphate dehydrogenase	[44]
*Linum usitatisumum L*.	Linaceae	Seeds	Reduces fasting blood sugar levels, total cholesterol; reduces carbohydrate absorption from gut and clinical symptoms of diabetes associated with dyslipidamia.	[20]
*Mangifera indica L*.	Anacardiaceae	Fruit, Leaves	Reduces glucose absorption in type 2 diabetes. Stimulates glycogenesis in liver causing reduction in blood glucose level.	[45]
*Morus alba L*.	Moraceae	Leaves	Antiphlogistic, diuretic, expectorant and antidiabetic. Increases the β-cell number in diabetic islets. Reduces levels of glycosylated hemoglobin. Decreases triglycerides, cholesterol and VLDL to normal levels in type II DM patients. Restores elevated levels of blood urea.	[46]
*Nerium oleander L*.	Apocynaceae	Leaves	Clorogenic acid, querecetin and cathechin induce post prandial hyperglycemia by acting as α-glucosidase inhibitors.	[21]
*Ocimum tenuiflorum L*.	Laminaceae	Leaves	Lowers blood glucose level, modulates cellular antioxidant defense system. Improves β cell function and enhances insulin secretion. Inhibits absorption of glucose from the intestine	[47]
*Piper nigrum L*.	Piperaceae	Seeds	Reduces glucose and serum lipid levels	[48]
*Terminalia chebula Retz*.	Combretaceae	Fruit	Decreases blood glucose levels by enhancing secretion of insulin from β cells of Langerhans or through extra pancreatic mechanism. Inhibits advanced glycosylation end products, which contribute to renal damage.	[49]
*Tinospora cordifolia (Willd.) Miers*	Menispermaceae	Stem	Decreases blood glucose level through glucose metabolism. It exhibits inhibitory effect on adrenaline-induced hyperglycemia.	[50]
*Trigonella foenum-graceum L*.	Fabaceae	Seeds	Decreases s post prandial blood glucose level.	[51]
*Zingiber officinale Rosc*.	Zingiberaceae	Rhizome	Lowers plasma glucose level	[52]

## Methods

### Material

Chemicals such as soluble starch, PPA (porcine pancreatic α-amylase), methanol, isopropanol, acetone, methyl-butyl-tertiary ether, cyclohexane and DMSO (dimethylsulfoxide) were purchased from SRL Pvt. Ltd, Mumbai, India. DNSA (3, 5-dinitrosalicylicacid) was obtained from HiMedia Laboratories, Mumbai, India. Acarbose was purchased from Sigma Aldrich, USA. All other chemicals procured were from local manufacturer and were of AR grade.

### Plant material

The above mentioned anti-diabetic plants were collected from the Western Ghats, Maharashtra State, India in the months of August-January. Herbarium specimen of each these plants was submitted to Botanical Survey of India, Pune for authentication. The voucher numbers are specified in additional file 1. Plant name, family, parts used along with its hypoglycemic and medicinal properties are listed in Table 1.

### Preparation of plant extracts

Air-dried plant material was finely crushed, powdered and successively extracted in polar to non-polar solvent on an increasing degree of non-polarity. The solvents used in sequential order were cold water, hot water, methanol, isopropanol, acetone, methyl-butyl-tertiary ether and cyclohexane. The significance of sequential extraction from polar to non-polar solvent is the fact that traditional Ayurvedic methods of preparing herbal formulations are mainly aqueous. It is also likely that peptides, proteins or glycans possessing α-amylase inhibitory activity would be extracted in aqueous system which would otherwise be denatured during organic solvent and high temperature extraction. Cold and hot water extracts were obtained by adding water to the crushed material in a ratio of 1:4 (w/v) and kept at 30°C (24 h) and 55°C (2 h) at130 rpm respectively. Each of the extract was filtered, centrifuged and the residue collected was subjected to subsequent solvent extraction. The organic solvents were added in a ratio of 1:3 (w/v) and refluxed with the residue for 3 h at their respective boiling temperatures. The extracts collected were filtered and stored at -20°C. Before assay, the organic filtrates were concentrated *in vacuo *in a rotary evaporator at 50°C while the aqueous extracts were lyophilized. Stock solutions for inhibition assay were prepared by dissolving up to 1.5 mg of each extract in 1 ml of DMSO and appropriately diluting it before use.

### Pancreatic α-amylase inhibition assays

#### Starch-Iodine colour assay

Screening of plant material for α-amylase inhibitors was carried out in a microtitre plate according to Xiao et al (2006) based on the starch-iodine test [23]. The total assay mixture composed of 40 μl 0.02 M sodium phosphate buffer (pH 6.9 containing 6 mM sodium chloride), 0.02 units of PPA solution and plant extracts at concentration from 0.1-1.5 mgml^-1 ^(w/v) were incubated at 37°C for 10 min. Then soluble starch (1%, w/v) was added to each reaction well and incubated at 37°C for 15 min. 1 M HCl (20 μl) was added to stop the enzymatic reaction, followed by the addition of 100 μl of iodine reagent (5 mM I_2 _and 5 mM KI). The colour change was noted and the absorbance was read at 620 nm on a microplate reader. The control reaction representing 100% enzyme activity did not contain any plant extract. To eliminate the absorbance produced by plant extract, appropriate extract controls without the enzyme were also included. The known PPA inhibitor, acarbose, was used a positive control at a concentration range of 6.5 - 32.8 μgml^-1^. A dark-blue colour indicates the presence of starch; a yellow colour indicates the absence of starch while a brownish colour indicates partially degraded starch in the reaction mixture. In the presence of inhibitors from the extracts the starch added to the enzyme assay mixture is not degraded and gives a dark-blue colour complex whereas no colour complex is developed in the absence of the inhibitor, indicating that starch is completely hydrolysed by α-amylase.

#### 3, 5-dinitrosalicylic acid assay

The inhibition assay was performed using the chromogenic DNSA method [24]. The total assay mixture composed of 500 μl of 0.02 M sodium phosphate buffer (pH 6.9 containing 6 mM sodium chloride), 0.04 units of PPA solution and extracts at concentration from 0.1-1.5 mgml^-1^(w/v) were incubated at 37°C for 10 min. After pre-incubation, 500 μl of 1% (v/v) starch solution in the above buffer was added to each tube and incubated at 37°C for 15 min. The reaction was terminated with 1.0 ml DNSA reagent, placed in boiling water bath for 5 min, cooled to room temperature, diluted and the absorbance measured at 540 nm. The control PPA at 0.21 Uml^-1 ^represented 100% enzyme activity and did not contain any plant extract. To eliminate the absorbance produced by plant extract, appropriate extract controls with the extract in the reaction mixture except for the enzyme were also included. The known PPA inhibitor acarbose was used a positive control at a concentration range of 6.5 - 32.8 μgml^-1^.

One unit of enzyme activity is defined as the amount of enzyme required to release one micromole of maltose from starch per min under the assay conditions.

The IC_50 _values were determined from plots of percent inhibition versus log inhibitor concentration and calculated by logarithmic regression analysis from the mean inhibitory values. The IC_50 _values were defined as the concentration of the extract, containing the α-amylase inhibitor that inhibited 50% of the PPA activity.

The other quantifiers were calculated as follows:

%   Relative enzyme activity=(enzyme activity of test/enzyme activity of control)*100.

%  inhibition in the α−amylase activity=(100−% relative enzyme activity).

#### Preliminary Phytochemical Analysis

The lead extracts were subjected to qualitative phytochemical analysis while the identification of the major phytoconstituents was carried out using GC-MS.

#### Qualitative phytochemical analysis

Lead extracts positive for α-amylase inhibition were tested for the presence of proteins, alkaloids, tannins, saponins, cardiac glycosides, steroids and flavonoids in accordance to Parekh and Chanda and Trease and Evans (1989) [25,26].

#### GC-MS Analysis

Analysis of the lead organic extracts exhibiting ≥50% inhibition on the PPA activity were performed on a GCMS-MS (HEWLETT PACKARD, GCD-1800 A) gas chromatograph equipped with DB 5 ms capillary column (30 m × 0.25 mm ID). Helium was the carrier gas with flow rate of 1 mL/min, the injector mode- split (1:60), the injection volume 1 μL, the temperature program used is as follows: 80°C (3 min), then increased to 280°C at 10°C/min, held at 280°C (10 min) and temperature scan, *m*/*z *35-300 amu. Appropriate solvent controls were also run. The identification of the components was based on the comparison of their mass spectra with those of NIST-Wiley 2008 library.

#### Statistical Data Analysis

All experiments were performed in 3 different sets with each set in triplicates. The data are expressed as mean ± SEM (standard error of the mean). Statistical analysis was performed for ANOVA (analysis of variance) followed by F test using SPSS version 11.5. Values of p which were ≤ 0.05 were considered as significant.

## Results and Discussion

Drugs that reduce post-prandial hyperglycaemia by suppressing hydrolysis of starch such as PPA inhibitors have been found useful in the control of diabetes mellitus [27,28]. Many herbal extracts have been reported for their anti-diabetic activities and are currently being used in Ayurveda for the treatment of diabetes. However, such medicinal plants have not gained much importance as medicines due to the lack of sustained scientific evidence.

In the present study, seventeen indigenous antidiabetic medicinal plants from the Western Ghats of India were screened for their α-amylase inhibitory potential. Plants used in the study along with the parts used and their hypoglycemic properties are listed in Table 1 while their voucher numbers are listed as an additional file 1. Several studies performed on these plants state them to be hypoglycemic, but none of these plants have been studied or tested for pancreatic α-amylase inhibitors in order to justify their hypoglycemic property. The rationale for performing extractions from polar to non-polar solvents is to confirm and validate the inhibitory activity in the aqueous extractions performed in the traditional manner as well as to search for newer, more potent inhibitory compounds in the organic solvents. Primary screening for α-amylase inhibition was performed based on starch-iodine colour complex formation. Upon extraction a total of 126 extracts were obtained from 17 plants. Of these 126 extracts screened, 21 extracts tested positive for PPA inhibition (Table 2). These lead extracts were further quantified with respect to PPA inhibition by chromogenic DNSA method and it was noted that 4 extracts exhibited low (< 10%) while 17 extracts showed significant (*p *value ≤ 0.05) PPA inhibitory activity (10 - 60.5%).

**Table 2:** Inhibition of PPA activity based on starch-iodine color assay by solvent extracts^a ^of different plants at 0.1-1.5 mgml^-1 ^(w/v)

Plant Species	Extracts						
	**CWE**	**HWE**	**ME**	**IPE**	**AE**	**MTBE**	**CHE**
*A. digitata *(Leaves)	-	-	-	-	-	-	-
*A. sativum *(Rhizomes)	-	-	-	-	-	-	-
*A. vera *(Leaf Gel)	+	-	-	-	-	-	+
*C. fistula *(Leaves)	+	+	+	+	-	+	+
*C. roseus *(Leaves)	-	-	-	-	-	-	-
*C. verum *(Bark)	-	-	-	-	-	-	-
*C. indica*. (Fruit)	-	-	-	-	-	-	-
*L. usitatisumum *(Seeds)	-	-	-	+	+	+	-
*M. indica *(Leaves)	-	-	-	-	-	-	-
*M. indica *(Fruit)	-	-	-	-	-	-	-
*M. alba *(Leaves)	-	-	+	+	-	-	-
*N. indicum *(Leaves)	-	-	-	-	-	-	-
*O. tenuiflorum *(Leaves)	-	-	-	+	-	-	-
*P. nigrum *(Seed)	-	-	-	-	-	-	-
*T. chebula *(Fruit)	-	-	-	-	-	-	-
*T. cordifolia *(Stem)	-	-	-	-	-	-	-
*T. foenum *(Seeds)	+	+	+	+	+	+	+
*Z. officinallis *(Rhizomes)	-	-	-	-	-	-	-
Acarbose (6.5 - 32.8 μgml^-1^)	+						

^a ^Extracts were sequentially prepared as described in Methods.

+: Inhibition

-: No Inhibition

CWE: Cold water Extract

HWE: Hot water Extract

ME: Methanol Extract

IPE: Isopropanol Extract

AE: Acetone Extract

MTBE: Methyl-tertiary-butyl Ether Extract

CHE: Cyclohexane Extract

**Figure 1:**
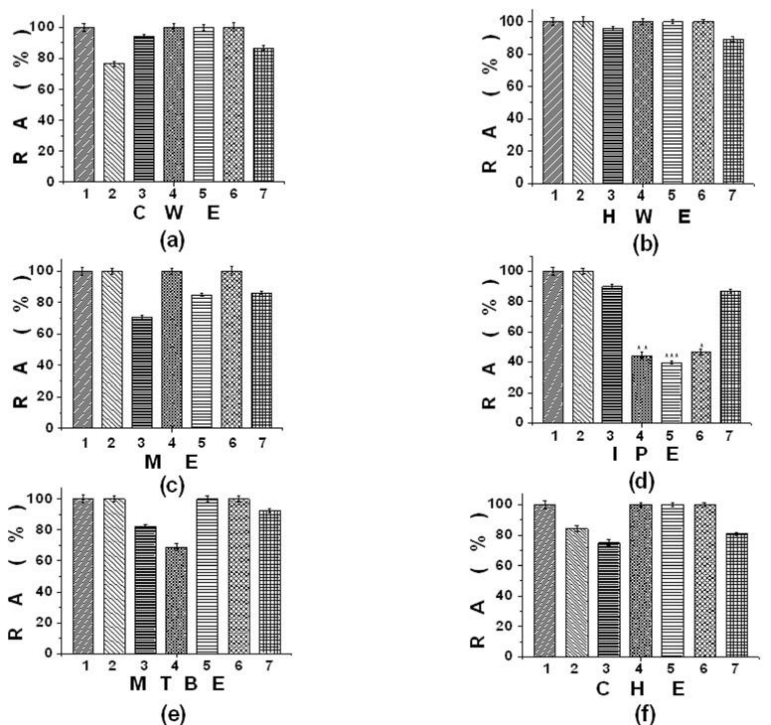
**The percent relative enzyme activity (RA %) of porcine pancreatic *α-*amylase (PPA) on inhibition with different extracts**. (a)Cold water extracts-CWE (b) Hot water extracts-HWE (c) Methanol extracts-ME (d) Isopropanol extracts-IPE (e) Methyl-tertiary-butyl-ether extacts-MTBE (f) Cyclohexane extracts-CHE of the listed plants. Pure porcine pancreatic α-amylase serves as control. The data is indicated as the mean ± SEM; (n = 3). The students F-test was used and the bars with different asterisks (***, **, *) show significant difference with respect to control (P < 0.05). 1 Control 2 *A. vera *3 *C. fistula *4 *L. usitatisumum *5 *M. alba *6 *O. tenuiflorum *7 *T. foenum*.

The aqueous extracts, i.e., cold water extracts of *A. vera*, and *T. foenum *exhibited 23.3%, and 13.4% inhibition at concentration of 2.5 mgml^-1 ^and 1.5 mgml^-1 ^respectively. Low inhibitory potential (*p *value ≤ 0.1) was observed for hot water extracts of *T. foenum *(10.8%) at a concentration of 3.5 mgml^-1^. Methanol extracts of *C. fistula *showed 29.2% PPA inhibition followed by *M. alba *(15.1%) and *T. foenum *(11.8%) at 1.9 mgml^-1^, 3.9 mgml^-1 ^and 5.2 mgml^-1^, respectively. Significant (*p *< 0.05) and strong PPA inhibition was observed for isopropanol extracts of *M. alba *(60.5%)at 1.8 mgml^-1^, *L. usitatisumum *(55.7%) at 0.65 mgml^-1 ^and *O. tenuiflorum *(53.4%) at 0.0094 mgml^-1 ^whereas low inhibition was noted in the case of *C. fistula *(10.0%) at 2.4 mgml^-1 ^and *T. foenum *(13.2%) at 3.6 mgml^-1^. Acetone extracts of *L. usitatisumum *(33.5%) exhibited moderate PPA inhibition (*p *value ≤ 0.05) at a concentration of 2.6 mgml^-1^. Methyl-tertiary-butyl ether extracts of *C. fistula *(18.9%) and *L. usitatisumum *(39.1%) showed PPA inhibitory activity at 3.4 mgml^-1 ^and 2.7 mgml^-1^, while cyclohexane extracts of *C. fistula *(25.2%), *T. foenum *(19.3%) and *A. vera *(15.8%) also showed moderate PPA inhibitory activity at 5.7 mgml^-1^, 1.9 mgml^-1 ^and 2.4 mgml^-1^, respectively (*p *value ≤ 0.1) (Figure 1). All the other extracts showed no significant PPA inhibitory activity (Table 2, Figure 1). None of the extracts were found to enhance PPA activity.

**Figure 2:**
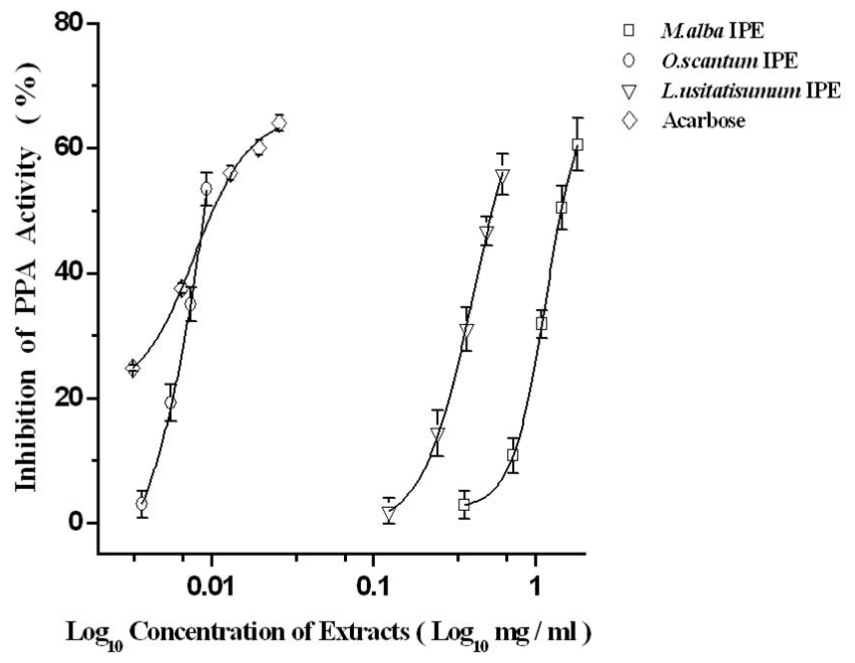
**The percent Porcine Pancreatic α-amylase inhibition (%) of different extracts at varying concentrations.** The data is indicated as the mean ± SEM; (n = 3). (P < 0.05). IPE - Isopropanol Extract.

Thus of the abovementioned extracts only three *viz*; *L. usitatisumum*, *M. alba *and, *O. tenuiflorum *isopropanol extracts exhibited strong i.e., ≥ 50% inhibition against PPA. Concentration dependent incubation of these isopropanol plant extracts of *L. usitatisumum*(0.5-2.6 mgml^-1^), *M. alba *(0.3-1.8 mgml^-1^) and *O. tenuiflorum *(0.001-0.009 mgml^-1^), respectively with α-amylase and starch *in vitro *resulted in a significant (*p *≤ 0.05) decrease in the enzyme activities from 0.21 U to 0.09 U. A dose dependent effect was observed on increasing the concentrations of the extract solution, suggesting a competitive type of inhibition. Plots of percent inhibition vs log concentration of extracts showed typical sigmoidal dose response curves (Figure 2). It was noted that the IC_50_, values i.e.,50% inhibition occurred at concentrations of 540 μgml^-1^, 1440 μgml^-1^, 8.9 μgml^-1 ^for *L. usitatisumum, M. alba, O. tenuiflorum*, respectively. Acarbose was taken as a positive control with an IC_50 _value at 10.2 μgml^-1^. *O. tenuiflorum *isopropanol extract exhibited an IC_50 _value less than acarbose suggesting that it could be a promising lead extract.

It could thus be speculated that these 3 extracts possess significant PPA inhibiting activity. Phytochemical analysis of these isopropanol extracts revealed the presence of alkaloids, steroids, saponins and cardiac glycosides in *L. usitatisumum*. Tannins, cardiac glycosides, flavonoids and saponins were detected in *M. alba *while *O. tenuiflorum extract *was found to contain alkaloids, tannins and flavonoids (Table 3).

**Table 3:** Qualitative phytochemical analysis of isopropanol extracts exhibiting ≥ 50% inhibition on PPA activity

Plant Species	*L. usitatisumum*	*M. alba*	*O. tenuiflorum*
Proteins	**-**	**-**	**-**
Alkaloids	**+**	**-**	**+**
Tannins	**-**	**+**	**+**
Cardic glycosides	**+**	**+**	**-**
Flavinoids	**-**	**+**	**+**
Saponins	**+**	**+**	**-**
Steroids	**+**	**-**	**-**

+ Detected

- Not detected

Preliminary GC-MS analysis based on retention time and molecular mass was performed to determine the nature of these phytoconstituents (Table 4). It was noted that in *L. usitatisumum *seed isopropanol extract the major compound detected could either be 2-cyclopentene-1-undecanoic acid (hydnocarpic acid) or cyclopentane undecanoic acid (dihydrohydnocarpic acid). The other compounds were in too low a concentration to be correctly identified. Such natural cyclic fatty acids are found in seeds from species of plants belonging to Flacourtiaceae family [29]. It has also been suggested that on heating of vegetal oils several fatty acids containing cyclopentnyl or cyclohexanyl ring are formed from linoleic and linolenic acids [30], under conditions similar to those used for solvent extraction. It has also been shown that compounds like linoleic and α-linoleic acids as well as lignin glycosides are present in *L. usitatisumum *seeds [31]. In our previous report, we have shown that *Linum usitatissimum *extract could significantly inhibit crude preparations of glycosidases isolated from murine pancreas but not from the liver or small intestines [32]. Hence, this extract seems to possess potential PPA inhibitory activity and could be a good candidate to carry out further *in vivo *studies.

**Table 4:** GCMS Profile of extracts exhibiting ≥ 50% inhibition on PPA activity

Name of the plant	Name of the compound	Molecular formula	Molecular weight	Area (%)	Retention time (min)
***O. tenuiflorum***	Camphene	C_10_H_16_	136.24	32.42	9.83
	Methyleugenol	C_11_H_14_O_2_	178.23	37.87	10.82
	2-heptanol, 5-ethyl	C_9_H_20_O	144.25	9.44	11.04
					
***M. alba***	Napthelene	C_10_H_8_	128.6	28.36	11.12
	Hexadeconoic acid	C_16_H_32_O_2_	256.4	40.76	14.23
	9, 12-octadecadienic acid	C_17_H_31_COOH	280.44	9.32	13.92
					
***L. usitatisumum***	2-cyclopentene-1-undecanoic acid/ cyclopentane undecanoic acid	C_16_H_28_O_2_ C_16_H_30_O_2_	252 / 254	85.6	21.03

The major phytoconstituents detected in the leaf isopropanol extract of *M. alba *were naphthalene, hexadeconoic (palmitic acid) and 9, 12-octadecadienoic acid (9,12-linoleic acid). These compounds have also been detected in *M.alba *leaves by earlier researchers [33]. Wei Song *et al *reported the presence of oxyresveratrol and 1-DNJ (1-deoxynojirimycin) in methanol extracts of *M.alba *leaves from China. 1-DNJ is a known inhibitor of α- glucosidase and α-amylase [34]. However, the methanol extract in our study showed relatively low PPA inhibitory activity (15.1%) as compared to the isopropanol extract (60.5%).

The major compounds identified from the leaf extract of *O. tenuiflorum*, were camphene, methyleugenol and 2-heptanol, 5-ethyl-. These monoterpenes, phenyl propanoids and alcohols have been reported earlier from *Ocimum *sp. [35-37]. Oral administration of ethanolic extracts of *O. tenuiflorum *were found to reduce serum glucose levels in normal and streptozotocin induced diabetic rats [38]. Essential oils obtained from *Cedrus libani* were found to inhibit α-amylase activity with an IC_50 _value of 0.14 mg/ml [39]. One of the compounds in this oil was identified as camphene, similar to that detected in *O. tenuiflorum*. Our earlier study [32] has also shown that *O. tenuiflorum *extracts possessed significant inhibitory activity against isolated glycosidase preparations obtained from murine pancreatic, liver and small intestinal tissues.

These findings suggest that one of the mechanisms by which *L. usitatisumum, M. alba *and *O. tenuiflorum *could be exhibiting their hypoglycemic effect would be by inhibition of PPA activity leading to retardation of starch hydrolysis, eventually lowering PPHG. All the phytoconstituents have earlier been reported from these plants but none of them have shown to possess PPA inhibitory activity. Since, the nature of the phytochemical responsible for PPA inhibitory activity has not yet been ascertained, the active principle(s) needs to be isolated and characterized through *in vitro *and *in vivo *studies. While some anti-diabetic plants in this study showed negligible inhibition against PPA, 17 extracts exhibited moderate to good enzyme inhibitory activity. Hence, though multiple mechanisms exist by which these plants could affect their hypoglycemic potential, one of the mechanisms by which some of these plants could be lowering PPHG levels could be due to PPA inhibition.

## Conclusions

Since the Indian population has long been using all the 3 plants for food and medicinal purposes, they form a part of the local pharmacopoeia. Our results suggest that one of the targets for hypoglycaemic property of *L. usitatisumum, M. alba *and *O. tenuiflorum *is pancreatic α-amylase inhibition. However, isolation and characterization of the compounds responsible for this inhibitory activity as well as *in vivo *studies need to be performed to confirm these observations. These phytochemical(s)/bioactive principle from the plants responsible for the PPA inhibition are currently being isolated and characterized.

## Competing interests

The authors declare that they have no competing interests

## Authors' contributions

SP performed the experiments as a part of her doctoral work. SSZ helped in the collection of samples and in their authentication. SB participated in the statistical analysis of the data and helped to draft the manuscript. All this work was carried out under the supervision of ARK who conceived and coordinated the study, designed the experiments and helped to draft the manuscript. All the authors have read and approved the final manuscript.

## Pre-publication history

The pre-publication history for this paper can be accessed here:

http://www.biomedcentral.com/1472-6882/11/5/prepub

## Supplementary Material

Additional file 1 **Plant Names and the Botanical Survey of India (BSI), Pune Voucher Numbers**. Click here for file
