# Supplementary material for: Potent α-amylase inhibitory activity of Indian Ayurvedic medicinal plants
Source: BMC Complement Altern Med. 2011 Jan 20;11:5. doi: 10.1186/1472-6882-11-5 (PMC3037352; doi:10.1186/1472-6882-11-5)
Supplement: Additional file 1 — Plant Names and the Botanical Survey of India (BSI), Pune Voucher Numbers. [file 1472-6882-11-5-S1.PDF]

Additional file: Plant names and their Voucher Numbers.

**Sr. No**	**Plant name**	**Voucher no.***
1	*Adansonia digitata L.*	SUPAADDI9
2	*Allium sativum L*	SUPAALSA8
3	*Aloe vera (L.)* Burm.f.	SUPAALVR12
4	*Casia fistula L.*	SUPACAF1
5	*Catharanthus roseus (L.)*. G. Don	SUPACARO2
6	*Cinnamomum verum* Persl	SUPACIV13
7	*Coccinia grandis (L.)* Voigt.	SUPACOGRA4
8	*Linum usitatisumum L.*	SUPALIUS15
9	*Mangifera indica L.*	SUPAMAIN11
10	*Morus alba L.*	PSMA1
11	*Nerium oleander L.*	SUPANERO10
12	*Ocimum tenuiflorum L.*	SUPAOCS14
13	*Piper nigrum L.*	SUPAPINI17
14	*Terminalia chebula Retz.*	SUPATECH6
15	*Tinospora cordifolia (Willd.) Miers*	SUPATICO17
16	*Trigonella foenum-graceum L.*	SUPATRIFO16
17	*Zingiber officinale Rosc.*	SUPAZINO5

***** The plants have been authenticated and deposited in Botanical Survey of India (BSI), Pune and the voucher numbers listed
